# Composite Score of Readiness (CSR) as Holistic Profiling of Functional Deficits in Footballers Following ACL Reconstruction

**DOI:** 10.3390/jcm10163570

**Published:** 2021-08-13

**Authors:** Łukasz Oleksy, Anna Mika, Aleksandra Królikowska, Maciej Kuchciak, Magda Stolarczyk, Renata Kielnar, Henryk Racheniuk, Jan Szczegielniak, Edyta Łuszczki, Artur Stolarczyk

**Affiliations:** 1 Orthopaedic and Rehabilitation Department, Medical University of Warsaw, 02-091 Warsaw, Poland; drstolarczyk@gmail.com; 2 Oleksy Medical & Sports Sciences, 37-100 Łańcut, Poland; 3 Polish Strength and Conditioning Association, 44-141 Gliwice, Poland; 4 Institute of Clinical Rehabilitation, University of Physical Education in Krakow, 31-571 Kraków, Poland; anna.mika@awf.krakow.pl; 5 Department of Sports Medicine, Faculty of Health Sciences, Wroclaw Medical University, 50-367 Wroclaw, Poland; aleksandra.krolikowska@umed.wroc.pl; 6 Department of Physical Education, University of Rzeszow, 35-959 Rzeszow, Poland; mkuchciak@ur.edu.pl; 7 Third Clinic of Internal Medicine and Cardiology, Medical University of Warsaw, 02-091 Warsaw, Poland; magda.stolarczyk@wum.edu.pl; 8 Institute of Health Sciences, Medical College of Rzeszow University, 35-959 Rzeszow, Poland; kielnarrenata@o2.pl (R.K.); eluszczki@ur.edu.pl (E.Ł.); 9 Institute of Physiotherapy, Faculty of Physical Education and Physiotherapy, Opole University of Technology, 46-020 Opole, Poland; h.racheniuk@po.edu.pl (H.R.); j.szczegielniak@po.edu.pl (J.S.)

**Keywords:** anterior cruciate ligament (ACL), composite score of readiness (CSR), injury prevention, rehabilitation, football, soccer

## Abstract

Background: The decision to return to sport (RTS) after anterior cruciate ligament (ACL) reconstruction is difficult; thus, coaching staff require a readable, easy-to-use, and holistic indication of an athlete’s readiness to play. Purpose: To present the Composite Score of Readiness (CSR) as a method providing a single score for RTS tests after ACL reconstruction. Methods: The study comprised 65 male football players (age 18–25 years), divided into three groups: ACL group—subjects after ACL rupture and reconstruction, Mild Injury (MI) group—subjects after mild lower limb injuries, and Control (C) group—subjects without injuries. The CSR was calculated based on three performed tests (Y-balance test, Functional Movement Screen, and Tuck Jump Assessment) and expressed as the sum of z-scores. The CSR index allows highlighting an athlete’s functional deficits across tests relative to the evaluated group. Results: The CSR indicated that relative to the group of athletes under the study, similar functional deficits were present. Comparing athletes following ACL reconstruction to both the MI and C groups, in the majority of subjects, the CSR index was below zero. The correlation between CSR and raw tests results indicated that the CSR is most strongly determined by YBT. Conclusion: The CSR is a simple way to differentiate people after serious injuries (with large functional deficits) from people without injuries or with only small deficits. Because the CSR is a single number, it allows us to more easily interpret the value of functional deficits in athletes, compared to rating those deficits based on raw tests results.

## 1. Introduction

Anterior cruciate ligament (ACL) injuries are very common in sports [1]. The most important goal for athletes after ACL reconstruction is a successful return to play [1,2]. It has been reported that from 78% to 98% of professional athletes, and 65% of amateurs, return to pre-injury level [3]. However, 74% of ACL re-injuries occur within the first 2 years [3]. After ACL reconstruction, the deficits were observed in postural stability as well as in alterations in knee and hip function. It was suggested that they might be associated with pathological movement patterns leading to further tissue overloads, and often, to ACL re-injury [4,5,6]. It has been suggested that return-to-sport (RTS) testing after ACL reconstruction should include several tests, such as isokinetic strength, hop test, and a jump landing task assessed with the Landing Error Scoring System or Tuck Jump Assessment (TJA) [6,7,8,9]. Additionally, movement patterns, mobility, and stability evaluation with the Functional Movement Screen (FMS) and dynamic balance via the Y-balance test (YBT) or the star excursion balance test were also recommended [9].

The decision to RTS after ACL reconstruction is difficult for clinicians to make. Moreover, the coaching staff require a readable, easy-to-use, and holistic indication of an athlete’s readiness to play [10]. This has raised a need to provide a single score to assess athletes’ RTS, rather than separately discussing each individual test result [10,11]. As we know, the athletes are regularly put through many tests whose individual results collected together produce an overwhelming amount of data [10,11,12]. Some authors propose identifying fewer but more predictive tests [8,13]. Others provide us with strategies that aim to reduce the data without decreasing the number of performed tests, such as Total Score of Athleticism (TSA), a single score of an athlete’s holistic athleticism introduced by Turner [10]. This way of assessment, by creating a single index, is already known in the literature. Such indices were used in gait evaluation [14,15,16,17]. The Gait Deviation Index (GDI), Gillette Gait Index (GGI), or Normalcy Index (NI) were derived to calculate the amount by which a subject’s gait deviates from an average normal profile and to represent this deviation as a single number [14,15,16,17]. The other index, called “Total Score of Athleticism (TSA)”, was described by Thurner et al. [10,11], and allowed coaches to examine the athleticism level of individual athletes relative to their teammates. This approach provided coaches with quick and easy-to-read data indicating how well each athlete performed in the tests relative to their teammates, and which areas are strengths, and which are weaknesses [10,11].

It is known that for coaches, most important is that the evaluation and interpretation of test results for a given athlete are clear and easy to interpret and that they are read in the same way by others. Direct reference to the baseline value (indicating a correct result) may allow for a precise assessment of the size of an athlete’s deficits. Following the TSA model, we would like to propose the injury risk index called “Composite Score of Readiness (CSR)”. This study is the first in which a single score index for RTS evaluation is described, which may differentiate athletes following a serious injury such as ACL reconstruction from athletes after mild musculoskeletal injuries and healthy controls. This index may allow assessing the level of functional deficits in these athletes. Therefore, the purpose of this study was to present the Composite Score of Readiness (CSR) as a method providing a single score for RTS tests after ACL reconstruction.

## 2. Methods

### 2.1. Participants

The studied participants involved 65 male football players belonging to regional teams participated in this study. Basing on a medical interview and gathered medical documentation, the players were divided into three groups, named consecutively “Group 1 (ACL)”, “Group 2 (MI)”, and “Group 3 (C)”. The three groups were similar in age, body weight, and body height, as presented in Table 1. Based on medical interviews and gathered medical documentation, they were free of the following diagnosed medical problems: currently experiencing pain and movement restriction, respiratory and circulatory system diseases, bilateral injuries in the lower limbs in the history, injuries of the trunk in the past, injuries of upper limbs in the past, and they gave consent to participate in research. The inclusion criteria in Group I were: clearance to play by an orthopedic specialist after primary unilateral ACL rupture and following arthroscopic reconstruction underwent during the 3 years before the research; bone–patellar, tendon–bone, or hamstring tendon autographs used during ACL reconstruction; no abnormalities and no history of injury in the contralateral knee; no to all of the following procedures: medial and/or lateral meniscectomy, medial and/or lateral meniscal transplant, posterior cruciate ligament repair, and medial or/and lateral collateral ligament repair/reconstruction osteoarthritis surgery in the ACL-reconstructed knee other than shaving; the lack of any upper limbs and trunk injuries in the past. The inclusion criteria in Group 2: clearance to play by an orthopedic specialist after grade I or “mild” lower limb muscle injury according to Grassi et al. [18] and following conservative treatment undergone during the 3 years before the research; no history of any other injuries in lower limbs, the lack of any upper limbs and trunk injuries in the past. The inclusion criteria in Group 3, a control group, was the lack of any lower and upper limbs and trunk injuries in the past.

Group 1 (ACL) (*n* = 24)—subjects after ACL rupture and reconstruction in previous 2–3 years who passed RTS including orthopedic and manual tests performed by a physiotherapist, muscle strength evaluation and hop tests, and were cleared to play (involved leg—after ACL reconstruction, uninvolved leg—contralateral limb without ACL injury);

Group 2 (MI) (*n* = 21)—subjects after mild lower limb injury in previous 2–3 years (involved leg—after mild injury, uninvolved leg—contralateral limb without injury);

Group 3 (C) (*n* = 20)—control group without injuries (the left limb was the equivalent of the involved limb and the right limb was the equivalent of the uninvolved limb).

The study participants were informed in detail about the research protocol and gave their written informed consent to participate in the study. Informed consent was acquired from the parent for participants under the age of 18. Approval of the Ethical Committee of Regional Medical Chamber in Kraków was obtained for this study (16/KBL/OIL/2016). All procedures were performed in accordance with the 1964 Declaration of Helsinki and its later amendments.

### 2.2. Procedures

A 5 min warm-up included general, non-specific exercises, which prepared the entire body for the performed tests. Then, each subject performed testing trials for each test to become fully familiar with the nature of the measurements. The protocol included the FMS test, the YBT, and TJA with 15 min intervals between the tests. An experienced researcher blinded to the subject group allocation performed all tests.

### 2.3. Functional Movement Screen

The FMS test (Functional Movement Systems Inc., Chatham, VA, USA), which includes assessment of body asymmetries and recognition of poor quality movement patterns, was performed according to the original methodology reported by Cook et al. [19,20,21]. FMS test inter-rater reliability was ICC = 0.87–0.89, and intra-rater was ICC = 0.81–0.91 [22,23].

### 2.4. Y-Balance Test

The YBT test (Move2Perform, Evansville, IN, USA) was conducted according to the criteria described by Plisky et al. [24,25]. Three reach trials were performed in each direction, first standing on the uninvolved leg and then on the involved (on the right leg and then on the left in the control group) [25]. The intra-rater reliability of the YBT was ICC = 0.85–0.91 and inter-rater reliability was ICC = 0.85–0.93 [25,26].

## 3. Tuck Jump Assessment

TJA was carried out according to previously described protocols [27,28]. Jumping efforts were recorded with the resolution 736 × 352 and 125 fps frame rate using the NiNOX 125 camcorder (NiNOX 125, Noraxon USA, Scottsdale, AZ, USA) from the sagittal and frontal plane view. Technique flaws were scored according to previously published form [28]. The reported TJA intra-tester mean percentage of exact agreement ranged between 87.2% and 100%, with kappa values of k = 0.86–1.0 [29].

### 3.1. Composite Score of Readiness (CSR)

The CSR was calculated from the 3 performed tests (FMS, YBT, and TJA) [10,11]. The CSR was the sum of z-scores, which represented the number of standard deviations by which the value of a raw score was above or below the mean of the measured variables. Raw scores above the mean have positive z-scores, while for those below the mean, the z-scores are negative. The z-scores were then summed to form a single score. Because z-scores and SD are unitless, the results can be summed across all tests. The CSR allows highlighting an athlete’s functional deficits across tests relative to the evaluated group. The interpretation of the CSR is based on the methodology described by Thurner et al. [10,11]. If zero represents the group average, any value above zero means that the athlete is better than average, while values below 0 indicate worse performance.

- The mean ± 1 SD contains ~68% of all test scores;
- The mean ± 2 SD ~95%;
- The mean ± 3 SD ~99%.

Two types of CSR were calculated. One type is the CSR, in which the z-score for individual athletes was calculated relative to their own group.

CSR_A_—calculated for athletes after ACL reconstruction relative to the group of athletes also after ACL reconstruction;

CSR_M_—calculated for athletes after mild lower limb injuries relative to the group of athletes with similar mild lower limb injuries;

CSR_H_—calculated for athletes without injuries relative to the group of similar athletes without injuries.

Then, the value of the CSR index for each athlete was converted relative to the remaining 2 groups. In this way, the relative CSR index was created, showing the size of the functional deficits of a given athlete in relation to different reference groups.

CSR_A-M_—calculated for athletes after ACL reconstruction relative to the group of athletes with mild lower limb injuries;

CSR_A-H_—calculated for athletes after ACL reconstruction relative to the group of athletes without injuries;

CSR_M-H_—calculated for athletes after mild lower limb injuries relative to the group of athletes without injuries.

### 3.2. Statistical Analysis

Statistical analysis was performed using STATISTICA 12.0 Pl software. All evaluated variables were reported as the arithmetic mean (x) and standard deviation (SD). The data were evaluated for normality with Shapiro–Wilk test. The *t*-test was used to determine the differences between groups. Additionally, Pearson’s linear correlation coefficient (r) was calculated (below 0.50—poor; between 0.50 and 0.75—moderate; between 0.75 and 0.90—good; above 0.90—excellent). Statistical significance was set at the level of (*p* < 0.05).

## 4. Results

**Figure 1:**
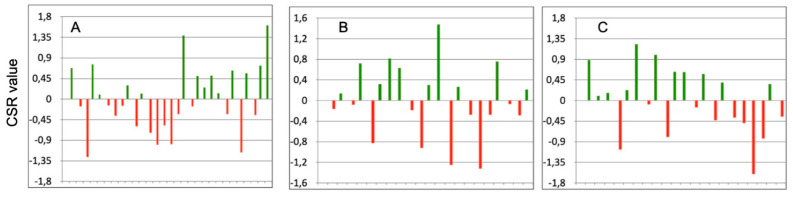
The values of CSR_A_ (**A**), CSR_M_ (**B**), and CSR_H_ (**C**) in athletes relative to the own group. Zero represents the group average CSR value; green bars indicate positive value (it means that the particular athlete is better than average); red bars indicate negative value (it means that the particular athlete is worse than average).

All CSR indices were normally distributed (*p* > 0.05). They are graphically presented in Figure 1, highlighting particular athletes’ performance relative to their group. Zero represents the team average in terms of CSR (sum of performed tests). Bars above the zero line represent athletes better than average, while bars below the line indicate worse than average athletes.

It has been shown that the CSR_A_ in 15 athletes was below zero (Figure 1A). The CSR_M_ was below zero in 11 athletes (Figure 1B), while the CSR_H_ in 10 athletes did not exceed zero (Figure 1C). This means that relative to the own group, athletes represented similar functional deficits. The CSR_A_, CSR_M,_ and CSR_H_ indices allow demonstrating who, within the group, exhibits better and who exhibits worse performance.

**Figure 2:**
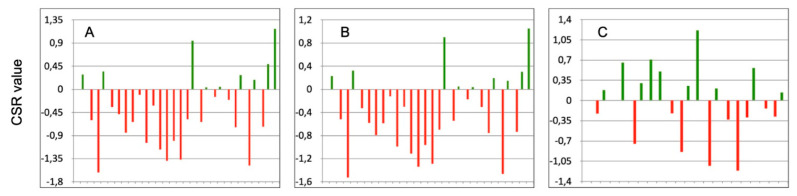
The values of CSR_A-M_ (**A**), CSR_A-H_ (**B**), and CSR_M-H_ (**C**) when comparing athletes after ACL reconstruction to both the MI and C groups. Zero represents the group average CSR value; green bars indicate positive value (it means that the particular athlete is better than average); red bars indicate negative value (it means that the particular athlete is worse than average).

Comparing athletes after ACL reconstruction to both the MI and C groups, it can be seen that in the majority of subjects, the CSR_A-M_ (Figure 2A) and CSR_A-H_ indices (Figure 2B) were below zero. It has been demonstrated that athletes with severe injury, similar to ACL reconstruction, despite passing the RTS, are in a functionally worse state compared to athletes with mild injuries or healthy ones. On the other hand, when comparing athletes with mild injuries to those healthy, they did not differ significantly from each other because the number of positive and negative CSR_M-H_ indices was similar (Figure 2C).

**Figure 3:**
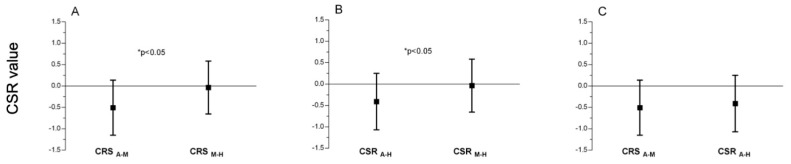
The difference between (**A**) CSR_A-M_ and CSR_M-H_; (**B**) between CSR_A-H_ and CSR_M-H_; (**C**) between CSR_A-M_ and CSR_A-H_.

The analysis of differences between the presented CSR indices showed similar results. The values of the CSR_M-H_ were significantly higher than CSR_A-M_ (Figure 3A). This difference was more visible when the CSR_A-H_ was compared to CSR_M-H_ and it was significant (Figure 3B). However, when CSR_A-M_ and CSR_A-H_ were compared, the values were similar, while the difference was non-significant (*p* > 0.05) (Figure 3C).

The correlation between CSR indices and raw test results indicated that the CSR is determined most strongly by YBT (Table 2). There was a strong and significant relationship between the composite score of the YBT test and each of the three CSR indices (r = 0.9–0.93) (Table 2). Interestingly, analyzing the correlation between the value of each CSR and the YBT components, it was noted that the relationship was good and significant for all reaching directions of both limbs but was stronger in the posterolateral and posteromedial directions (r = 0.79–0.85). The anterior direction demonstrated a moderate relationship (r = 0.57–0.64). Both the FMS and TJA tests presented a much weaker relationship with the CSR indices. The relationship between the FMS composite score and CSR was poor (r = 0.35–0.38). Between the TJA composite score and the CSR, the correlation was also poor and non-significant (r = 0.30–0.35) (Table 2).

## 5. Discussion

The most important information from this study is that the CSR is a simple way to differentiate athletes with severe functional deficits following serious injuries such as ACL rupture and reconstruction from those without injuries or with only small functional deficits. This difference may be expressed as one index, which is easy to interpret. We have also noted that athletes after ACL reconstruction are in a functionally worse state than athletes following mild injuries or those who are healthy.

Unfortunately, as indicated by some authors, passing the RTS criteria did not result in a decreased risk of ACL re-injury, suggesting that some functional deficits are still present and may not be diagnosed during evaluation [30,31]. The RTS may include many tests, and there is no consensus as to which are the most sensitive and valid. Moreover, many of them may be difficult to rate unambiguously, especially when a number of tests are performed by the athlete and provide non-homogenous results. Very often, coaches and athletes are not interested in the raw score of the test, which may be difficult for interpretation, but they require a simple indication of an athlete’s readiness-to-play after ACL reconstruction.

This manner of assessment, through a one-number index, has been discussed in the literature on the subject of gait pattern disorder assessment [14,15,16,17] or in the evaluation of athletes’ sports level [10,11]. The single number reflects the value by which a subject’s gait deviates from the average for normal gait [14,15,16,17]. Additionally, the TSA is a method providing a single score of holistic athleticism, from fitness tests and informing how well someone performed relative to the others taking part in the test [10,11]. Therefore, we suggest that the CSR may be a good tool for properly discriminating athletes with high functional deficits after serious injuries such as ACL rupture and reconstruction from athletes with mild injuries or from healthy ones. The CSR may allow to easily rate the severity of post-injury functional deficits in relation to the reference value (the value close to zero). According to the methodology described by Thurner et al. [10,11], a score of one indicates that an athlete has obtained a better score than 84% of his/her group, while a score of two indicates a score better than 97%.

In the present study, the CSR_A_, CSR_M,_ and CSR_H_ indices allowed to show who, within the group, demonstrates better and who demonstrates worse performance. As was presented, when compared athletes with similar functional deficiencies, those with greater and those with smaller deficits can be distinguished. However, based on this type of CSR, we do not know the size of these deficits in relation to the normal values, e.g., in healthy controls without injuries. Only by comparing athletes after ACL reconstruction to other reference groups (healthy or mildly injured) can the size of these deficits be observed. In our study, by comparing athletes post-ACL reconstruction to both the MI and C groups, it could be seen that the majority exhibited CSR indices below zero. It was shown that athletes with severe injuries, such as ACL reconstruction, despite passing the RTS, were in a functionally worse state compared to those with mild injuries or healthy individuals. On the other hand, comparing athletes with mild injuries to healthy subjects, they did not differ significantly from each other because the amount of positive and negative indices was similar.

It has been recommended that RTS testing after ACL reconstruction should incorporate several tests, but it is unclear which are most associated with a successful RTS [8,9]. The TJA was reported to identify players at risk of ACL injuries by identifying side-to-side asymmetries, neuromuscular imbalances, as well as jumping and landing technique flaws [7]. The FMS has been suggested as an effective tool during RTS evaluation [24], while its limited usage has been underlined by others [32,33]. Chorba et al. [34] have reported that the FMS was unable to differentiate between subjects who had not experienced any ACL rupture and those who had. Moreover, it was shown that ACL injuries alter movement patterns, but the FMS test is unable to detect them, and these deficits may increase the risk of re-injury [35]. Additionally, the application of YBT in RTS screening provides equivocal results. Some researchers have indicated the usefulness of YBT [36,37,38], while others have not found any differences in YBT scores among athletes following ACL reconstruction who were or were not cleared for return to unrestricted activity [39]. In the current study, it has been reported that CRS calculated from FMS, YBT, and TJA tests indicated that athletes after ACL reconstruction are in a functionally worse state than those following mild injuries or without injuries.

A crucial issue, which was also underlined by Thurner et al. [10,11], is that the diagnostic value of such an index may be influenced by the kind of applied test as well as by the amount (number) of the tests from which z-scores are summed. They have reported that the validity of the TSA index was largely determined by the relevance of the implemented fitness tests [10,11]. This problem may also be a weakness of the CSR index. Therefore, we have analyzed which test influenced the final index the most; thus, in other words, which test is most indicative of functional deficits related to ACL reconstruction. Our results allowed us to suggest that CSR is determined most strongly by YBT. Interestingly, analyzing the correlation between the value of each CSR and the YBT components (not only a composite score), it was noted that the relationship was good and significant for all reaching directions of both limbs but was stronger in the posterolateral and posteromedial directions. The anterior direction (considered by many authors to be the most deficient in people after ACL) showed a moderate relationship. On the other hand, both the FMS and TJA tests presented a much weaker association with the CSR indices. This means that these tests are less indicative for assessing the size of functional deficits in athletes after ACL reconstruction. However, this issue requires further research to see which tests are the most sensitive in detecting specific deficiencies post-ACL reconstruction. Another critical aspect that requires further research is the analysis of individual test components because, as has been shown in our research, the composite score of a given test does not always provide the same information as its individual elements. Perhaps, most optimal would be an index created from parts of the tests and not only from their composite results.

This study also has some limitations which should be addressed. We calculated the CSR only from three tests (FMS, YBT, TJA). Perhaps, if more tests would be included in CSR calculations, its diagnostic value would be more substantial, and the CSR itself would be more comprehensive. Additionally, because psychological factors have emerged as important in the RTS process, it seems required to include psychological tests as a component of CSR. Therefore, there is a need for future research, including a broader selection of tests. Moreover, the utility of CSR should be assessed in future studies concerning its reliability, sensitivity to rehabilitation interventions, and predictability of RTS outcomes.

## 6. Conclusions

The CSR is a simple way to differentiate individuals following serious injuries (with large functional deficits) from those without injuries or with only small deficits. Because the CSR is a single number, it allows easier interpretation of the functional deficit size in athletes than if rating those deficits from raw tests results.

## Figures and Tables

**Table 1:** Subjects characteristics.

	Group 1	Group 2	Group 3
Number of subjects (*n*)	24	21	20
Height (cm)	175 ± 4	177 ± 6	178 ± 6
Weight (kg)	77.3 ± 7.6	74.3 ± 9.1	75.8 ± 8.8
Age	22.7 ± 3.6	20.5 ± 3.7	23.1 ± 2.8

No significant difference was found for any variable.

**Table 2:** The relationship between CSR and raw tests results.

Outcome Measure	Side	CSR_A_	CSR_A-M_	CSR_A-H_
FMS Composite Score (points)		0.38 *	0.35	0.38 *
TJA Composite Score (points)		0.36	0.30	0.35
YBT Composite Score (%)	I	0.92 *	0.93 *	0.92 *
	U	0.90 *	0.92 *	0.90 *
YBT Anterior Reach (%)	I	0.56 *	0.60 *	0.57 *
	U	0.59 *	0.64 *	0.59 *
YBT Posterolateral Reach (%)	I	0.85 *	0.83 *	0.85 *
	U	0.83 *	0.83 *	0.84 *
YBT Posteromedial Reach (%)	I	0.82 *	0.82 *	0.81 *
	U	0.80 *	0.79 *	0.79 *

(I) Involved side; (U) uninvolved side. CSR_A_—index calculated for athletes after ACL reconstruction relative to the group of athletes also after ACL reconstruction. CSR_A-M_—index calculated for athletes after ACL reconstruction relative to the group of athletes with mild lower limbs injuries. CSR_A-H_—index calculated for athletes after ACL reconstruction relative to the group of athletes without injuries. (TJA) Tuck Jump Assessment; (FMS) Functional Movement Screen; (YBT) Y-balance test. Values are expressed as Pearson correlation coefficient (r). * *p* < 0.05.

## Data Availability

All data generated or analyzed during this study are included in this published article.
